# Cardiometabolic reprogramming drives right ventricular dysfunction in pulmonary arterial hypertension: Novel mechanisms and therapeutic implications

**DOI:** 10.1016/j.chmed.2026.05.004

**Published:** 2026-05-08

**Authors:** Saihu Liu, Mari Okazaki, Yanling Wu

**Affiliations:** a Key Laboratory for Traditional Chinese Korean Medicine Research (State Ethnic Affairs), Key Laboratory of Natural Medicines of the Changbai Mountain (Ministry of Education), College of Pharmacy, Yanbian University, Yanji 133002, China; b Laboratory of Pharmacology, Faculty of Pharmaceutical Sciences, Josai University, Saitama 350-0295, Japan

**Keywords:** cardiometabolic reprogramming, mitochondrial dysfunction, pulmonary arterial hypertension, right ventricular dysfunction, single-cell multi-omics technology

## Abstract

With the understanding on right ventricular (RV) dysfunction in pulmonary arterial hypertension (PAH), the perspective innovatively integrates research paradigms on targeting cardiometabolic reprogramming of RV protective therapies to break through the bottleneck of current PAH treatment. The research focuses on three core areas: analyzing and validating metabolic dysregulation in the RV of PAH, exploring cell-specific metabolic dysregulation in the heart of PAH, and probing into the therapeutic strategy for PAH targeting RV metabolism. These aspects aim to deepen the scientific understanding of cardiometabolic reprogramming of RV dysfunction in PAH. This work provides a theoretical foundation and experimental evidence for developing RV-directed therapies that complement existing PAH treatments and improve patient outcomes.

## 1 Introduction

Pulmonary arterial hypertension (PAH) is a fatal disease characterized by a progressive increase in circulatory resistance of the pulmonary artery system. As pulmonary vascular disease worsens, the right ventricular (RV) is subjected to unprecedented pressure load, gradually transitioning from adaptive hypertrophy to decompensated dilation and failure. It is worth noting that RV failure is the most important determinant of prognosis in PAH patients, but its treatment options are extremely limited (Hemnes et al., 2024a). In recent years, beyond the traditional perspective of hemodynamics and systolic function, the cardiometabolic reprogramming, the fundamental changes of energy signaling networks for cardiomyocytes facing chronic stress, has been revealed as the core pathological mechanism driving the RV from compensation to decompensation. This provides a crucial perspective to understand the essence of RV failure and develop new treatment strategies.

## 2 Metabolic reprogramming of RV dysfunction: Core shift in research paradigm of PAH

Metabolic reprogramming of RV dysfunction is not only the key mechanism of PAH induced heart failure, also opens up a new and promising field of targeted therapy for PAH. Typically, 60%−90% of the energy in adult myocardial cells comes from fatty acid oxidation (FAO), while the rest comes from glucose and lactate oxidation, which is an efficient way of energy production. Under the pressure overload caused by PAH, the metabolic pattern of RV cardiomyocytes undergoes pathological changes, known as metabolic reprogramming, characterized by enhanced glucose metabolism, inhibited FAO, mitochondrial dysfunction, and substrate utilization rigidity (Flam & Arany, 2023).

### 2.1 Enhanced glucose metabolism

Glucose metabolism begins with glycolysis, producing pyruvate that, under aerobic conditions, is converted to acetyl-CoA by pyruvate dehydrogenase (PDH). Under normal circumstances, glucose is completely burn through aerobic oxidation in mitochondria, producing a large amount of adenosine triphosphate (ATP) energy. In the diseased RV, even under aerobic conditions, cardiomyocytes turn to the less efficient and primitive glycolysis, only performing preliminary breakdown of glucose (Ryan & Archer, 2014). This metabolic shift, similar to the Warburg effect of cancer cells, leads to a sharp decrease in ATP generated per glucose, causing the heart to fall into an energy famine and unable to maintain normal contractions, thereby accelerating heart failure. This shift is accompanied by increased expression of glucose transporters glucose transporter 1 (Glut1) and Glut4, as well as glycolytic enzymes such as hexokinase 2 (HK2) and phosphofructokinase. PDH is a key enzyme for aerobic oxidation, and its activity determines metabolic flow. PDH kinase (PDK) inhibits PDH activity, and its overactivity in PAH will trigger metabolic reprogramming.

### 2.2 Inhibition of FAO

The healthy myocardium mainly transports fatty acids into the mitochondria, efficiently providing energy. In the depleted RV, the key gate responsible for transporting fatty acids and the core assembly line responsible for breaking down fatty acids, such as carnitine palmitoyltransferase 1 (CPT1) and medium-chain acyl-CoA dehydrogenase (MCAD), become scarce and inactive. This results in the ineffective utilization of fatty acids, and the interruption of the advanced energy supply to myocardial cells, further exacerbating the energy crisis (Namazi, Eftekhar, Mosaed, Shiralizadeh Dini, & Hazrati, 2024).

### 2.3 Mitochondrial dysfunction

In the metabolic reprogramming process of RV dysfunction, mitochondrial dysfunction is the core executive link of energy crisis. This is manifested as the destruction of mitochondrial cristae structure, decline in biosynthetic ability, and steady state imbalance of division and fusion, collectively leading to damage to the quality and integrity of mitochondria. The fundamental functional consequence is oxidative phosphorylation uncoupling, which means that the proton gradient generated by electron transfer is inefficiently dissipated and cannot drive ATP synthesis. This series of systematic breakdowns in structure, quantity, and function leads to the degradation of mitochondria from efficient energy factories to inefficient organelles that produce reactive oxygen species, directly causing severe depletion of ATP production necessary for myocardial contraction and survival.

### 2.4 Substrate utilization rigidity

For metabolic reprogramming of RV dysfunction, substrate utilization rigidity refers to the loss of the ability of myocardial cells to dynamically and flexibly switch between major energy metabolism pathways, such as glucose oxidation and FAO, according to physiological needs due to sustained mechanical and metabolic stress. This is not simply change for energy metabolism, but a systemic metabolic dysfunction caused by inhibition of core metabolic regulatory network function, comprehensive decline in mitochondrial quality and function. As a result, myocardial cells are forced to lock in an inefficient and high consumption metabolic mode (usually glycolysis). Even if there are other available substrates, they cannot be effectively utilized. The loss of metabolic flexibility severely weakens the ability of RV to respond to fluctuations in energy demand, thereby accelerating the irreversible transition from compensatory hypertrophy to decompensated failure. The substrate utilization rigidity caused by the RV of PAH is mainly due to the metabolic switch failure driven by the PDK/PDH axis, which is regulated by multiple signals such as forkhead box O1 (FOXO1), hypoxia-inducible factor-1*α* (HIF-1*α*), and Randle cycle. In clinical, the evaluation of substrate utilization rigidity mainly relies on positron emission tomography (PET) and emerging hyperpolarized magnetic resonance spectroscopy imaging (HP MRSI). PET can macroscopically display an increase in glucose uptake, while HP MRSI can specifically detect PDH activity, providing powerful tools for evaluating the condition and guiding treatment.

Based on existing evidence, the four metabolic phenotypic characteristics of RV metabolic reprogramming do not exist in isolation, and their importance, temporal sequence, or causal relationships present a complex network rather than a simple linear chain. The general consensus is that metabolic reprogramming does exist, but its specific patterns exhibit significant heterogeneity in patients and disease stages. Mitochondrial dysfunction is the core and the basis for all subsequent changes. Enhanced glucose metabolism is a key executor of pathological remodeling. This is one of the most distinct and consistent characteristics of the RV of PAH. Both animal models and patients have observed an increase in RV myocardial glucose uptake and glycolysis. This directly leads to an increase in lactate production, which has become a core feature of RV failure and can even be detected in patients’ plasma, potentially becoming a biomarker for monitoring disease progression. Inhibition of FAO is an important consequence, particularly evident in the late stage of decompensation. Substrate utilization rigidity is the ultimate functional result of these transformations. In short, metabolic reprogramming in PAH with RV dysfunction is a dynamic and heterogeneous process. This heterogeneity emphasizes the necessity of personalized metabolic intervention strategies based on patient specific genetic backgrounds and clinical phenotypes in the future.

## 3 Cross cellular metabolic reprogramming of RV dysfunction: Critical turning point from phenomenon description to mechanism analysis and precise intervention

### 3.1 Characteristics and interactions of cardiomyocytes with metabolic dysregulation

The pathological state of the RV is the synergistic action of different types of cardiomyocytes, and their metabolic patterns are also different. As the contraction unit of the heart, the metabolic imbalance of cardiomyocytes is the core of functional decline. Its metabolic reprogramming characteristics include decreased FAO, increased glycolysis, mitochondrial dysfunction, and possibly increased ketone body utilization (Bornstein, Tian, & Arany, 2024). Thus, these directly cause a decrease in contraction force, lack of energy, and excessive lactate production, which affects the microenvironment. The metabolic reprogramming characteristics of cardiac fibroblasts include significantly enhanced glycolysis and active glutamine metabolism. These provide biological materials for activation and proliferation, promoting collagen synthesis, and leading to myocardial fibrosis. The metabolic reprogramming of immunocytes, such as macrophages, is characterized by pro-inflammatory subtype (M1) mainly through glycolysis, and the repair subtype (M2) relies on oxidative phosphorylation (Swirski & Nahrendorf, 2018). Their metabolic state determines inflammatory phenotype, driving or alleviating myocarditis and fibrosis. The metabolic reprogramming of cardiac endothelial cells is mainly characterized by glycolysis, possible increasing fatty acid synthesis. These will affect angiogenesis and barrier function, and its metabolites can signal regulate other cells.

These cells do not operate in isolation. For example, lactate released by cardiomyocytes can be taken up by cardiac fibroblasts, promoting their activation. While activated cardiac fibroblasts affect the metabolism of cardiomyocytes and immunocytes by secreting cytokines. Such metabolic dialogue constitutes the complex network of RV metabolic reprogramming.

### 3.2 Revolutionary perspective of single-cell multi-omics technology

Traditional research cannot distinguish the contributions of different cardiomyocytes in cardiometabolic reprogramming of RV dysfunction. Single-cell multi-omics techniques, such as single-cell transcriptomics and spatial transcriptomics, have solved this problem by accurately depicting the metabolic profiles and dialogues of RV cardiomyocytes, fibroblasts, immune cells, and other cells (Rong & Zhou, 2025). By utilizing these technologies, researchers have been able to identify cell specific metabolic states, decipher intercellular metabolic dialogues, and reconstruct regulatory networks. In the discovery of diseases, different cell subpopulations have unique metabolic gene expression and metabolite profiles. Through spatial transcriptome, it is possible to locate which cells are producing large amounts of lactate, and which neighboring cells are absorbing and utilizing this lactate to form the lactate shuttle. Integrating multiple omics can reveal key transcription factors and signaling pathways that drive metabolic reprogramming of RV dysfunction, such as the HIF-1*α* and AMP-activated protein kinase (AMPK)/peroxisome proliferator-activated receptor *γ* coactivator 1*α* (PGC-1*α*) pathways.

Single-cell multi-omics technology could systematically reveal the metabolic reprogramming mechanism of the RV of PAH. Traditional analysis considers tissues as homogeneous mixtures, while single-cell multi-omics technology can accurately distinguish different cell types, such as cardiomyocytes, endothelial cells, and fibroblasts to decipher cell heterogeneity and draw cell metabolic maps. A multi-omics study used a bone morphogenetic protein receptor 2 (BMPR2) mutant mouse model to systematically depict the specific changes in multiple pathways of RV failure, including glycolysis, fatty acid metabolism, and tricarboxylic acid (TCA) cycle (Hemnes et al., 2024b, Li et al., 2023, Lu et al., 2020). The lactate was the only metabolite that was elevated in both the RV and plasma, suggesting that it could serve as a potential biomarker for RV dysfunction. Single-cell multi-omics is used to identify key metabolic regulatory genes and driving cells, and sprouty1 (SPRY1) is identified as a novel key biomarker in the PAH pathogenesis, which is closely related to endothelial cell angiogenesis metabolic reprogramming. Single-cell sequencing can also reveal a broader metabolic regulatory network, lactylation modification is generally elevated in PAH tissues through integrated analysis.

From a perspective, the study of RV metabolic reprogramming across cell types marks a new era of spatial resolution, cell specificity, and dynamic interaction. Future breakthroughs will rely on the deep integration of single-cell multi omics technology, computational biology, and novel disease models. The ultimate goal is to identify key nodes that can be intervened in from complex metabolic networks, bringing new precision metabolic therapies to the clinical challenge of RV dysfunction.

## 4 Therapeutic potential of targeted cardiometabolic reprogramming of RV dysfunction in PAH

### 4.1 Cardiometabolic reprogramming regulators

Based on the above mechanism, cardiometabolic reprogramming regulators have become a promising strategy for improving RV dysfunction in PAH (Mansoor & Ibrahim, 2025). Targeting PDK to restore PDH activity and normal energy metabolism is an emerging strategy for treating RV dysfunction in PAH. PDK inhibitors, such as dichloroacetate (DCA), aim to relieve the inhibition of PDH, restore pyruvate to the TCA cycle, improve mitochondrial glucose oxidation, and enhance ATP production efficiency. Preclinical studies have shown that DCA can improve RV function. Enhance FAO could reduce lipid accumulation and reverse RV dysfunction. Peroxisome proliferator-activated receptor *γ* (PPAR*γ*) agonist pioglitazone, *L*-carnitine supplementation, or CPT-1 agonists reduce lipid accumulation and reverse RV dysfunction, which is related to increased CPT1 expression and enhanced FAO. Dietary intervention targeting glutamine and serine aims to improve pulmonary vascular stiffening, remodeling, and downstream in PAH. The glutamine antagonist 6-diazo-5-oxo-*L*-norleucine and glutaminase 1 inhibitor CB-839 could reduce RV hypertrophy, enhance cardiac output cardiac fibrosis, and inflammatory cell infiltration in rodent models.

### 4.2 Multi-target and multi-pathway modes of natural products

Natural products show the unique therapeutic advantages of in regulating RV metabolic reprogramming. Natural products can restore the metabolic imbalance between glycolysis and fatty acid oxidation, which is the core feature of metabolic reprogramming in PAH induced RV dysfunction. Natural products can bidirectional precisely regulate this metabolic imbalance. On the one hand, they enhance FAO by upregulating the PPAR*α*/PGC-1*α* signaling axis, such as ursolic acid and paeoniflorin (Gao et al., 2020). On the other hand, they reduce excessive glycolysis by inhibiting the pyruvate kinase M2 (PKM2)/Glut1/lactate dehydrogenase A (LDHA) axis, such as shikonin and kaempferol (Li et al., 2023). This bidirectional correction regulation mode is difficult to achieve for most single target chemically synthesized drugs. The root cause of RV metabolic reprogramming lies in mitochondrial dysfunction, including reduced mitochondrial biosynthesis, decreased oxidative phosphorylation efficiency, mitochondrial kinetic imbalance, and mitochondrial autophagy defects. Natural products achieve metabolic integration regulation centered on mitochondrial quality control and functional optimization from different dimensions, such as chrysin promotes mitochondrial biosynthesis through PPAR*γ* upregulation, resveratrol enhances mitochondrial oxidative function through sirtuin 1 (SIRT1) activation. Natural products often have strong antioxidant and anti-inflammatory activities while regulating metabolic pathways. For example, *α*-carvacrol reduces hypoxic oxidative damage through the nitric oxide (NO)-soluble guanylyl cyclase (sGC)-cyclic guanosine phosphate (cGMP) pathway, while rhodiola extract reduces FAO metabolic toxicity by inhibiting acylcarnitine levels (Budziak, Kloza, Krzyżewska, & Baranowska-Kuczko, 2025).

The core of clinical treatment for the RV of PAH is to reduce RV afterload, optimize preload, and enhance myocardial contractility, while traditional Chinese medicine mainly serves as an auxiliary means to improve the overall state through multi-target intervention. The main therapeutical principles of traditional Chinese medicine in the RV of PAH are activating blood and resolving stasis, nourishing *qi* and resolving phlegm, and promoting diuresis and reducing swelling. Its advantage lies in the overall regulation of multiple components and targets. The current evidence mostly comes from small sample clinical observations or basic research, but it demonstrates positive potential, such as Qili Qiangxin Capsules and Linggui Zhugan Decoction (Lu et al., 2020, Lv et al., 2024). The essence of traditional Chinese medicine lies in the principle of syndrome differentiation and treatment, which is highly compatible with the concept of individualized treatment pursued by modern medicine. The personalization of combination therapy is not only reflected in drug selection, but also in the individualization of dosage form, treatment intensity, and treatment timing. However, there is still a lack of high-quality large-scale clinical trials, which hinders their widespread application. More rigorous research is needed in the future to clarify the specific plans, applicable populations, and long-term benefits of combining traditional Chinese medicine with personalized treatment.

Interventions targeting a single metabolic target often trigger compensatory cellular feedback mechanisms and weaken long-term efficacy. The multi-target and multi-pathway modes of natural products can simultaneously act on multiple nodes of glucose metabolism, fatty acid metabolism, mitochondrial function, oxidative stress to form the synergistic network effect and effectively avoid the limitations of compensatory feedback on therapeutic efficacy.

The current main strategies for clinical treatment of PAH mainly target pulmonary vasodilation, with limited intervention in RV metabolic reprogramming, and often accompanied by adverse reactions such as liver function damage, headache, and hypotension. Natural products are mostly derived from traditional medicinal plants showing safety in long-term clinical applications. They have a wider treatment window and are suitable for chronic PH patients who require long-term medication.

Natural products have shown promising prospects in the field of RV metabolic reprogramming regulation caused by PAH. However, the focus should be on large-scale clinical trials to verify safety and optimize dosing regimens, precise identification and selection of metabolic targets, and optimization of lead compounds based on natural product structures. With the rapid development of multi-omics techniques and systems biology methods, the therapeutic potential of natural products in regulating RV metabolic reprogramming is expected to be fully explored and clinically translated.

## 5 Clinical translation barriers

Advancing RV metabolic protection therapy to clinical application involves not only scientific challenges but also multidisciplinary hurdles in engineering on imaging metabolic probes, biopsy risks, and species differences, etc. As the most commonly used in clinical, ^18^F-fluorodeoxyglucose (^18^F-FDG) PET cannot distinguish between glucose oxidation and glycolysis. Moreover, high myocardial uptake in physiological conditions can obscure lesion signals. ^11^C-palmitate and 14-(*R*,*S*)-[^18^F]fluoro-6-thia-heptadecanoic acid (^18^F-FTHA) could accurately track fatty acid oxidation, ketone bodies, and glutamine metabolism, however their extremely short half-life or complex synthesis requirements. Then, RV biopsy can easily lead to perforation and pericardial tamponade due to thin RV wall, often less than 5 mm. With minimal tissue acquisition, it is impossible to distinguish the metabolic characteristics of cardiomyocytes, fibroblasts, and macrophages. The metabolic reprogramming of non-cardiomyocytes, especially metabolically active macrophages and myofibroblasts, which may be the key driver of myocardial fibrosis. RV specific exosomes in plasma might be used to analyze the metabolic enzymes, metabolites, or noncoding RNA as the alternative approach. Also, establishing single-cell metabolic profiles by single cell omics will contribute to infer circulating biomarkers in clinical stages. Another, PAH animal models often fail to perfectly replicate the progressive, heterogeneous, and complex pathological features of human PAH. Human PAH is a slowly progressing and dynamically fluctuating loads, accompanied by multiple impacts such as chronic hypoxia and neurohormonal activation. It may lead that the effective metabolic regulators in animal models fail in complex human environments.

Faced with the above challenges, the most feasible path in the short term is to screen for PAH subgroups with high risk of metabolic decompensation through liquid biopsy and non-invasive imaging. Also, it is necessary to invest in metabolic phenotype research in large animal models.

## 6 Research significance

**Fig. 1:**
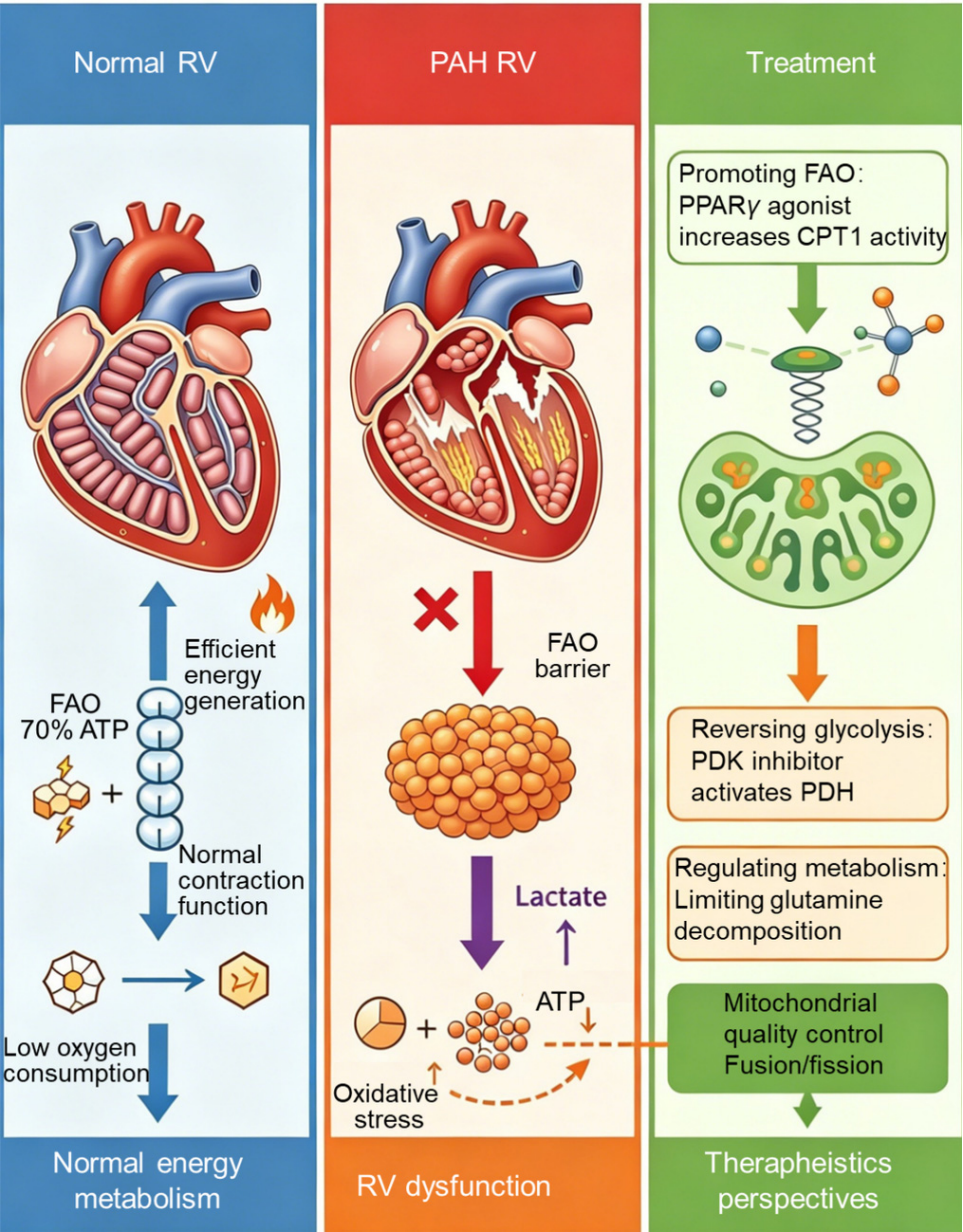
Metabolic reprogramming in RV dysfunction in PAH.

RV metabolic reprogramming is the key pathogenic link in the transition of PAH from adaptive hypertrophy to failure (Fig. 1). It connects hemodynamic stress with cellular dysfunction, inflammation, and fibrosis. By deeply understanding this process and actively developing RV protective therapies targeting the metabolism of both cardiomyocyte and non-cardiomyocyte, it is expected to break through the current bottleneck of PAH treatment and truly improve the long-term prognosis of patients. In the past, the PAH treatment mainly focused on the pulmonary blood vessels themselves, such as dilating blood vessels and inhibiting vascular remodeling. But now it has been found that the RV functional status is the key to determining the outcome of disease. The traditional paradigm centered on blood vessels but ignored the metabolic mechanism of the RV transitioning from compensatory hypertrophy to failure. The new paradigm emphasizes the equal importance of both blood vessels and RV, which means not only treating pulmonary vascular disease, but also actively protecting the RV, especially by intervening in the metabolic processes of myocardial and non-myocardial cells to prevent heart failure. This marks a fundamental shift in the paradigm of PAH research from vascular centric to equal emphasis on both blood vessels and ventricles. The ideal solution for the future may be a combination strategy of vasodilators and RV metabolic regulators. At the same time, identifying specific RV metabolic phenotypes of patients through metabolomics and imaging techniques, such as positron emission tomography-computed tomography (PET-CT) will achieve individualized treatment plans. Despite the broad prospects, translating metabolic targeted therapy into clinical practice still faces challenges. It is necessary to clarify the specific spatiotemporal characteristics of metabolic reprogramming in human PAH, verify the long-term safety and effectiveness of RV specific metabolic interventions, develop non-invasive techniques for assessing RV metabolic status to guide treatment.

## CRediT authorship contribution statement

**Saihu Liu:** Methodology, Formal analysis, Writing – original draft. **Mari Okazaki:** Supervision, Conceptualization, Writing – review & editing. **Yanling Wu:** Conceptualization, Resources, Supervision, Funding acquisition, Writing – original draft, Writing – review & editing.

## Declaration of competing interest

The authors declare that they have no known competing financial interests or personal relationships that could have appeared to influence the work reported in this paper.
